# Food Composition Data and Tools Online and Their Use in Research and Policy: EuroFIR AISBL Contribution in 2022

**DOI:** 10.3390/nu14224788

**Published:** 2022-11-12

**Authors:** Alessandra Durazzo, Siân Astley, Maria Kapsokefalou, Helena Soares Costa, Angelika Mantur-Vierendeel, Loek Pijls, Luca Bucchini, Marija Glibetić, Karl Presser, Paul Finglas

**Affiliations:** 1 CREA—Research Centre for Food and Nutrition, Via Ardeatina 546, 00178 Rome, Italy; 2 EuroFIR AISBL, Rue Washington 40, 1050 Brussels, Belgium; 3 Department of Food Science & Human Nutrition, Agricultural University of Athens, 11855 Athens, Greece; 4 Department of Food and Nutrition, National Institute of Health, 1649-016 Lisbon, Portugal; 5 Loekintofood, 3524 GG Utrecht, The Netherlands; 6 Hylobates, Consulting S.R.L., 00135 Rome, Italy; 7 Centre of Research Excellence in Nutrition and Metabolism, Institute for Medical Research-National Institute of the Republic of Serbia, University of Belgrade, 11129 Belgrade, Serbia; 8 Premotec GmbH, 8400 Winterthur, Switzerland; 9 Quadram Institute Bioscience, Norwich NR4 7UQ, UK

**Keywords:** EuroFIR AISBL, food data banks, nutrients, bioactive compounds, standardization, harmonization, interoperability

## Abstract

Food, nutrition, and health are linked, and detailed knowledge of nutrient compositions and bioactive characteristics is needed to understand these relationships. Additionally, increasingly these data are required by database systems and applications. This communication aims to describe the contribution to databases and nutrition fields as well as the activities of EuroFIR AISBL; this member-based, non-profit association was founded to ensure sustained advocacy for food information in Europe and facilitate improved data quality, storage, and access as well as encouraging wider exploitation of food composition data for both research and commercial purposes. In addition to the description of its role and main objectives, a snapshot of EuroFIR AISBL’s activities over the years is also given using a quantitative research literature analysis approach. The focus of this communication is to provide descriptions and updates of EuroFIR’s online tools, i.e., FoodEXplorer, eBASIS, and PlantLIBRA, by highlighting the main uses and applications. Integrating food-related infrastructures and databases, following standardized and harmonized approaches, and considering interoperability and metrological principles are significant challenges. Ongoing activities and future plans of EuroFIR AISBL are highlighted, including, for instance, work within the Food Nutrition Security Cloud (FNS-Cloud) to make food, nutrition, and (food) security data more findable, accessible, interoperable, and ultimately reusable.

## 1. Introduction

### 1.1. Food Databases and Nutrition: The Current Context

Research exploring relationships between diet and health have, in recent decades, garnered increasing interest in biologically active components in foods alongside nutrients. In addition to nutritional function, bioactive components of the diet have potentially beneficial health properties, which has led to greater perception of foods as functional ingredients or nutraceuticals. Moreover, new properties attributed to nutrients, and the interactions between nutrients and bioactive compounds, are also being explored. Food, nutrition, and health are linked, but detailed knowledge of nutrient compositions and bioactive characteristics is needed to understand these connections, and data characterizing bioactive compounds are required.

In this context, the development of specialized databases for components with nutritional and nutraceutical properties, as well as updating food composition databases (FCDBs) and publishing other specialized datasets (e.g., plant botanicals), at national and European levels, to supply knowledge that can help reduce the burden of chronic diseases and adopt sustainable nutrition patterns, is a challenge [1,2,3,4].

Food composition compilers aim to produce, collect, and present data in standardized formats to “speak a common language”: this allows comparisons across national databases and fosters exchange and collaboration among countries [5,6]. Simultaneously, researchers are publishing databases compiling information about metabolites in humans and identifying novel dietary biomarkers.

Databases comprised of nutrients, bioactive compounds, metabolites, or food supplements are essential tools for understanding human nutrition and public health and are vital resources for nutritionists, dietitians, food developers, and researchers, with a range of different applications, e.g., dietary assessment, exposure studies, food labeling, epidemiological studies, clinical research, nutritional education, and support for food industries and SMEs for nutrient labeling and health claims. These databases are exploited in epidemiology, food production and nutraceutical, pharmaceutical, and therapeutic interventions, and research trends are frequently redefined.

Initial construction of a dataset for nutrients, bioactive compounds, or compounds classes, and their inclusion in a specialized database, should be monitored to ensure approaches are standardized and database functionalities harmonized with existing resources. Moreover, updating and expanding existing databases, as more comprehensive resources, should be encouraged, perhaps through certification. Databases dedicated to particular or characteristic categories of foods are also valuable (e.g., traditional and ethnic foods, and recipe databases). Traditional and ethnic foods should also be included in national FCDBs and recipe collections. These foods constitute an important part of culture, history, identity, heritage, and local economy of a region or country and are key elements in the dietary patterns of each country [7,8].

Databases dedicated to bioactive compounds, particular individual classes of compounds, as reported by Scalbert et al. [9], can fail to reflect numbers and diversity of chemical features, range of dietary sources, variability from one source to another, and different procedures used to extract compounds as well as analytical techniques used. Additional factors that should be considered are that (i) only a few compounds within a class are investigated, and (ii) there is a lack of appropriate well-documented analytical methods [9] for application in food research.

Technological advances that allow management of “big data”, management of distributed and secured data using blockchain or process data using natural language processing, algorithms, or artificial intelligence are relatively new in the exploitation of food composition data. Nevertheless, technologies, tools, and infrastructures are now emerging with properly orchestrated processes leading to delivery of more findable, accessible, interoperable, and reusable (FAIR) big data ecosystems [10,11,12,13,14,15].

In this context, this communication aims to describe the contribution of the international, member-based, non-profit association EuroFIR AISBL to the status of FCDBs and related information being published in Europe and beyond.

### 1.2. EuroFIR AISBL: Role, Organization, and Main Features

The mission of EuroFIR AISBL is to promote harmonization and exploitation of high-quality food composition data and foster cooperation and participation in development with national compiler organizations. EuroFIR AISBL coordinates activities with experts and national compilers, contributing to worldwide efforts to produce and maintain high-quality food information, datasets and tools.

EuroFIR AISBL was formed in 2009, arising from the European Food Information Resource (EuroFIR) Network of Excellence (Grant agreement ID: 513944) and NEXUS project (2005–2013, Grant agreement ID: 265967) [16], to ensure sustained advocacy for food information in Europe and beyond in partnership with FAO INFOODS, and facilitate improved data quality, storage and access, and reuse for research and commercial purposes.

To provide a brief snapshot of the research history and status related to the contribution of EuroFIR to food information databases and nutrition fields, a quantitative literature analysis was carried out on 6 June 2022 using Scopus (https://www.scopus.com/home.uri, accessed on 6 June 2022). The search string “EuroFIR” was used, and bibliographic data (i.e., year, count, document type, origin, institutions, etc.) were recorded. Scopus functions “analyze” and “create citation report” were utilized for basic analyses. The search returned 101 documents covering the period 2005–2022, and the main subject areas were *Agricultural and Biological Sciences*, *Nursing*, *Medicine*, and *Chemistry*.

The oldest work was published by McKevith, B. in the journal *Nutrition Bulletin* during 2005 and describes working towards a European food information resource—EuroFIR, but also more specifically FCDBs or tables to be used by dietitians and health professionals, food manufacturers and producers, and other researchers; keywords for this paper were database; European Commission; food composition; and food information resource [17]. Further works, published in 2006, were “EuroFIR update—One pagers and web features” [18], as well as a paper published by the network describing development of a comprehensive, coherent, and validated food composition databank in Europe for nutrients [19]. The most cited work was by Trichopoulou et al. [20], where the importance of including traditional foods in current national FCDBs was highlighted. Papers identified in the search, distributed by typology mainly included, “articles” (74.3%), “reviews” (9.9%), “conference papers” (6.9%), and “book chapters” (3%) (data from Scopus database). Two documents belonging to “editorial” category were also reported, one dedicated to the Second International EuroFIR Congress 2007 [21], and the other to the 3rd International EuroFIR Congress 2009 [22].

Limiting the search to documents including “EuroFIR” as a keyword identified 35 publications, the most recent of which was published by Westenbrink et al. [23] and focused on EuroFIR activities to improve harmonization of documentation for aggregated/compiled values in FCDBs. Kapsokefalou et al. [5] described challenges related to quality of food composition data with a particular emphasis on needs in the Mediterranean area. Machackova et al. [24] published guidelines for calculating nutrient contents of foods by calculation for food business operators. Some works published in 2016 addressed (i) EuroFIR quality approaches for managing food composition data [25]; (ii) implementation of EuroFIR document and data repositories as accessible resources of food composition information [26]; and (iii) GAMA-EuroFIR guidelines for the assessment of methods of analysis [27].

**Figure 1:**
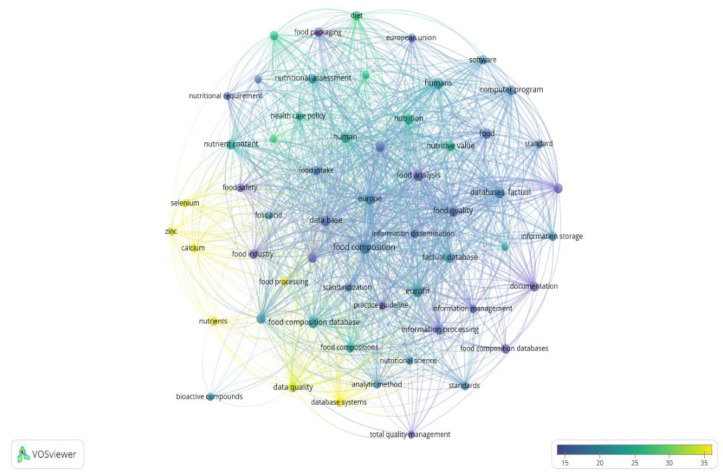
Term map for EuroFIR activities. Bubble size represents numbers of publications. Bubble color represents citations per publication (CPP). Bubbles are closer to one another if terms co-appeared more frequently (bibliometric data were extracted from Scopus and elaborated using VOSviewer software).

The “full records and cited references” (document title, citation counts, abstract, author, and index keywords) were exported and processed using VOSviewer software (version 1.6.16, 2020; www.vosviewer.com, accessed on 6 June 2021) [28,29,30]. In total, 58 terms were identified and are visualized as a term map in Figure 1. Figure 1 allowed for the identification of terms correlated with research related to EuroFIR activities, and existing research focused on these topics. Among recurring keywords, food composition, food analysis, Europe, food composition database/s, database (factual)/factual database, human/s, data base, food quality, nutrition, nutrition value, information processing, food, quality control, data quality, nutrient content, nutritional assessment, reference database, food composition data, documentation, food intake, food packaging, food industry, diet, information storage, database system, software, and dietary intake appeared most.

## 2. Updates and Results of EuroFIR AISBL Activities on Implementation of EuroFIR AISBL’s Food Data Banks

**Figure 2:**
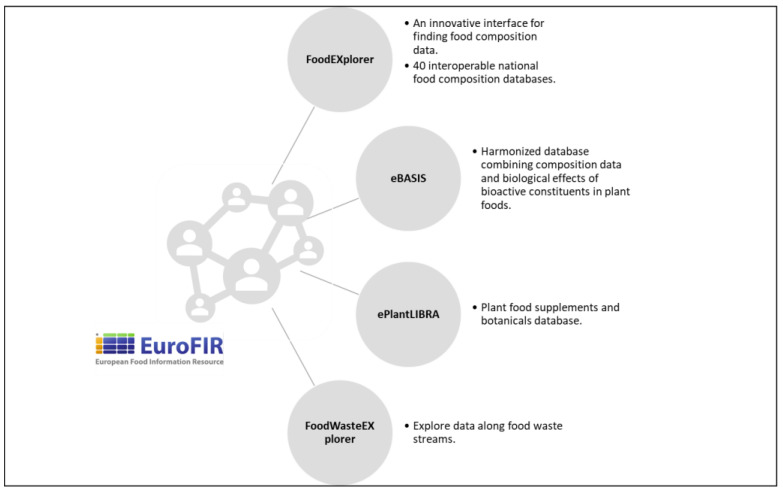
Representation of main EuroFIR AISBL tools.

EuroFIR AISBL provides a resource at the European level for compilers and user communities through online tools, e.g., FoodEXplorer, eBASIS, PlantaLIBRA, FoodWasteEXplorer [1] (https://www.eurofir.org/our-tools/, accessed on 26 October 2022) (Figure 2).

eBASIS, ePlantLIBRA and FoodWasteExplorer are based on data from peer-reviewed literature evaluated critically by experts. National FCDBs, which form part of FoodExplorer, are based mostly on experimental data and follow EuroFIR compilation guidelines. All FCDBs included in FoodEXplorer are based on a quality assessment system. EuroFIR also set up technical working groups that continue to progress underpinning topics, such as documentation, branded food datasets, recipe calculation, laboratory analysis, and use of FoodCASE for managing food composition data (https://www.eurofir.org/discussion-groups/, accessed on 26 October 2022).

Description of EuroFIR AISBL’s Food Data Banks is organized in two subsections: the subsection *EuroFIR’s Approaches* gives an overview of: (i) quality management system and standard operating procedures; and (ii) food description and classification systems, while the subsection *EuroFIR AISBL’s Food Data Banks: Main features and updates* describes functionalities, updates and use of FoodEXplorer, eBASIS, ePlantLIBRA, and FoodWasteEXplorer.

### 2.1. EuroFIR AISBL’s Approaches

Many international projects and research networks have tried to standardize methods for collection, management, and publication of food data. Efforts in the development of procedures to define and establish standardized collections of food composition data, specifically nutrient content, have also been carried out (e.g., description, selection, preparation, references, analytical or computational approach, compilation) [5,31,32]. EuroFIR AISBL, along with national compilers, have put considerable effort, now recognized globally, in establishing standardized and harmonized food datasets to assure the quality of both compilation processes and their presentation [1,2,5,23,24,25,26,27,33,34,35,36,37,38,39,40,41,42,43,44].

#### 2.1.1. Quality Management System and Standard Operating Procedures

To ensure the conformity (interoperability) of FCDBs, datasets must demonstrate transparency in aggregation, validation, and compilation based on standardized documentation and quality evaluation. EuroFIR AISBL has established a quality-data management system and harmonized and standardized processes.

EuroFIR AISBL’s standard operating procedures (SOPs) are identified in various publications such as: (i) documentation of foods, nutrients, and background information (i.e., metadata); (ii) attribution of quality indices to original data; (iii) coding of original data before data entry; (iv) quality check on original data coding and data entry; (v) physical storage of original data; (vi) selection of original data for aggregation; (vii) selection and application of algorithms to produce aggregated and compiled datasets; (viii) validation of aggregated and compiled datasets; and (ix) selection of aggregated and compiled datasets for dissemination as a published database or tables as well as guidelines for quality data evaluation [23,45,46].

Documentation of information concerning foods, components, values, and references is essential in maintaining a FCDB. One working groups developed guidelines for default value documentation of aggregated/compiled values using the EuroFIR AISBL’s standard and thesauri. Options for aggregation/compilation in the FoodCASE data management system were taken as the starting point [23].

#### 2.1.2. Food Description and Classification Systems

There is a consensus on the importance of nomenclature, (food) descriptions, and classification of foods. In this context, and with a view to the exchange of data, design, and development of a database primarily requires exact identification of a food. FoodEx2 is a standardized food classification and description system developed by EFSA, and supported by FAO INFOODS, to describe characteristics of foods and food supplements in exposure assessment studies. This system comprises flexible combinations of classifications and descriptions based on a hierarchical system for food safety-related domains (i.e., food consumption, contaminants, pesticide residues, veterinary drug residues, zoonoses–biological and microbiological aspects, botanicals, and food composition) [47,48,49,50,51].

LanguaL^TM^ or “Langua aLimentaria” or “Language of food” (LanguaL^TM^) is generally recognized as a method for describing foods, facilitating the capture and exchange of food data. More specifically, LanguaL^TM^ has a controlled vocabulary for systematic food descriptions that can be used with thesauri for faceted classification [52]. As described by Møller and Ireland [53], any food (or food product) can be described systematically using a combination of characteristics. In turn, these characteristics can be categorized and coded for computer processing, and resulting viewpoint/characteristic codes can be used to retrieve data about foods from similarly coded external sources. Each food is described using a set of standard, controlled terms taken from facets characteristic of the nutritional and/or quality aspects of a food, such as: food source, i.e., ANIMAL USED AS FOOD SOURCE [B1297], PLANT USED AS FOOD SOURCE [B1347]; cooking, i.e., TOASTED [G0010], BOILED [G0014], STEAMED WITH PRESSURE [G0022], DEEP-FRIED [G0029]; preservation methods, i.e., PASTEURIZED BY IRRADIATION [J0119], PRESERVED BY FREEZING [J0136], PRESERVED BY STORAGE IN CONTROLLED ATMOSPHERE [J0176]; and treatment applied, i.e., BLEACHED [H0197], PUFFED [H0268], EXTRUDED [H0352]. Several applications of simple foods, food preparations, recipes, food supplements, and agro-food wastes have been carried out [54,55,56,57].

LanguaL^TM^ was developed principally to support data exchange, whereas FoodEx2 was developed as a food classification and description system for exposure and risk assessment studies, i.e., exposure to contaminants. LanguaL^TM^ codes are assigned following a facet scheme set in advance, which defines and describes foods (i.e., source, origin, physical state, heat treatment, cooking method, treatment, preservation, packaging, etc.), and this scheme must be applied and maintained for all food items. FoodEx2 coding aggregates food products according to need without following a pre-agreed scheme. For instance, POACHED EGGS are codified by FoodEX2, revision 2 [47,48,49,50,51] using a single base term [A032D], whereas LanguaL^TM^ codifies them using terms string: 02 EGG AND EGG PRODUCTS (EUROCODE2) [A0725], HEN [B1713], WHOLE EGG WITHOUT SHELL [C0225], WHOLE, SHAPE ACHIEVED BY FORMING [E0147], FULLY HEAT-TREATED [F0014], SIMMERED, POACHED OR STEWED [G0020], HUMAN CONSUMER, NO AGE SPECIFICATION [P0024]. Recently, examples of applications using both systems on food preparations and recipes were given by Durazzo et al. [54]. FoodEx2 uses implicit descriptors to reduce code length, whereas LanguaL^TM^ descriptors are fully explicit and structured. Both LanguaL^TM^ and FoodEx2 are updated regularly based on feedback from users. User training courses are run for both LanguaL^TM^ and FoodEx2.

LanguaL^TM^ and FoodEx2 are the main food description and classification systems, and both are well developed, widely used, and recognized at European and International levels [42]. Their use also represents the likely direction of future work, specifically the automation of matching, mapping, and data quality checking. Consequently, maintenance and updating of both systems must be carried out regularly through exchanges between users and developers, considering evolution of the food market and new food classification needs in different applications. Subsequently, the correct application of classification and description systems relies on standard operating procedures (SOPs), regular updates, and multi-disciplinary cooperation [42].

These schemes are, however, not the only coding approaches, and their use can be supplemented with other systems such as ontologies. FoodOn is an open source, harmonized, and comprehensive food ontology that supports global food traceability, quality control, and data integration [58,59]. It is composed of term hierarchy facets that cover basic raw food source ingredients; process terms for packaging, cooking, and preservation; and an upper-level variety of product type [58,59]. For nutrient composition, and likely also bioactives and botanicals, however, EuroFIR AISBL recommends ongoing activities use of LanguaL^TM^ and FoodEx2 [42].

### 2.2. EuroFIR AISBL’s Food Data Banks: Main Features and Updates

#### 2.2.1. FoodEXplorer

FoodEXplorer [1] is an innovative interface for searching simultaneously food composition data in most publicly available national FCDBs in the European Union (EU) Member States as well as Canada, the United States, New Zealand, and Japan. Currently, FoodEXplorer [60] host 40 interoperable national FCDBs (EuroFIR AISBL FoodExplorer, https://www.eurofir.org/foodexplorer/foodgroups.php, accessed on 6 June 2022). Food and nutrient data are linked throughout LanguaL^TM^.

For the search, “access African and EMR data” (https://www.eurofir.org/FoodEXplorer/foodgroups.php?data=D2, accessed on 6 June 2022) was also created, in addition to “access on FoodEXplorer”. Open (publicly available) datasets have been developed and published for Australia and New Zealand, Iran, Iraq, Kuwait, Morocco, Pakistan, South Africa, and Tunisia, supported by projects including EMR (Eastern Mediterranean Regional data, funded by UK Medical Research Council Global Challenges Research Fund in collaboration with the World Health Organization’s Eastern Mediterranean Regional Office), African data (funded by the UK Biotechnology and Biological Sciences Research Council Global Challenges Research Fund in collaboration with the FAO INFOODS AFROFOODS network), and Food Standards Australia New Zealand (funded by the Commonwealth of Australia and Food Standards Australia New Zealand, 2018).

In this regard, it is worth mentioning the work of Ene-Obong et al. [61], which describes the importance and use of reliable food composition data by nutrition/dietetic professionals in solving Africa’s nutrition problems and focuses on constraints and the roles of FAO INFOODS and AFROFOODS as well as other stakeholders in future initiatives. The authors noted how AFROFOODS recommended that compilation, dissemination, and use of food composition tables (FCTs)/FCDBs should be given priority and included in country and regional development and investment plans. Similarly, AFROFOODS has called on governments to incorporate food composition into curricula for higher education, particularly nutrition and dietetics professional learning, but also health and agriculture [61]. More recently, EuroFIR AISBL and Quadram Institute Bioscience (QIB, UK) have assisted AFROFOODS in capacity building and development of a website with help from Premotec GmbH (PMT, CH)—a Swiss company experienced into implementation of software solutions for food data, i.e., food composition, food consumption and total diet studies—to increase visibility and enhance networking, and development of a road map for future activities.

In 2019, analysis of harmonized EuroFIR documentation for macronutrient values in 26 European FCDBs was carried out by Westenbrink et al. [41] to evaluate the impact of harmonized documentation and its usefulness for research and/or policy; documentation of most properties describing nutrient values was complete, even if the percentage coded as unknown varied from 14% to 49% for value and method types, method indicator, and acquisition type. The same authors reported some inconsistencies and incomplete information (about 65% missing) in coding and documentation [41]. Additionally, they noted how easy data exchange was supported by harmonized procedures for data documentation according to EuroFIR guidelines, even if comparability of carbohydrate, dietary fiber, protein, and energy values remained difficult due to multiple definitions and formulae, particularly lack of details about analytical and calculation methods [41].

A potential solution to improve harmonization was defined and published in 2020 in EuroFIR FoodEXplorer Standard [42], providing updated guidelines for collecting, compiling, and updating food composition data. In particular, the following actions were proposed for datasets being uploaded to FoodEXplorer: (i) before uploading, EuroFIR will standardize data units; (ii) energy will be re-calculated using European labelling legislation EU Regulation No. 1169/2011 (https://bit.ly/3g5yegE, accessed on 26 October 2022) recommendations and presented as both kcal and kJ; and data on less common components, such as polyols, organic acids, and salatrims, should be provided and included in recalculation; (iii) vitamin A will be presented as retinol activity equivalents (RAE); (iv) for calculated components, only one value per component ID will be shown; and (v) the use of both LanguaL^TM^ food description coding and FoodEx2 classification and description coding is recommended but not mandatory [42]. In 2020, following user feedback, functionalities of FoodEXplorer were updated, specifically: (i) advanced search functionalities; (ii) formatting of downloads for Excel; (iii) options for sorting components; (iv) presentation of component values and documentation; and (v) selection of foods for comparison.

Elaborations and applications using data from FoodExplorer were carried out among users and compilers. An example of a FoodEXplorer application for creating specialized food composition datasets, in this case for vitamin D in foods based on European standards for dietary intake assessment, was described by Milešević et al. [62] while Gurinović et al. [63] elaborated development, functionalities, and application of DIET ASSESS & PLAN (DAP) software, a platform for standardized and harmonized food consumption collection, comprehensive dietary intake assessment, and nutrition planning to support public health nutrition research in Central Eastern European Countries (CEEC). DAP enabled exploitation of national FCDBs from FoodEXplorer and their exploration using other online tools [63].

Another example of the utilization of data from FoodExplorer was given by FishChoice 2.0 (www.fishchoice.eu, accessed on 26 October 2022) [64]. FishChoice 2.0 is a tool, relaunched by Marquès et al. [64] as a tool for consumers and nutrition professionals, which delivers information about health benefits/risks as well as some sustainability information for fish and seafood on an individual basis, based on calculation of nutrients and contaminant intakes [64]; FoodEXplorer was used to collect nutrient data for fish and seafood species typically consumed in Europe for inclusion in FishChoice 2.0 [64].

#### 2.2.2. eBASIS—Bioactive Substances in Food Information System

Demand for easily accessible information on composition, intakes, and activities of bioactive compounds is significant among researchers. Bioactive Substances in Food Information System (eBASIS) [65] is a web-based database containing scientifically validated information describing the composition of bioactive compounds in major European plant foods. eBASIS was launched in 2006 [66,67] as a user-friendly, efficient, and flexible interface for the scientific community and food industry. It was the first EU harmonized database combining composition data and biological effects for compound classes, including polyphenols, isoflavones, glucosinolates, phytosterols, glycoalkaloids, and xanthine alkaloids, in 15 languages [68,69].

EuroFIR eBASIS was compiled using data from the peer-reviewed literature evaluated critically by experts. Tutorials for users are available online (https://www.eurofir.org/our-tools/ebasis/, accessed on 26 October 2022) as well as via a short video demonstrating how eBASIS can be used (*Introduction to eBASIS*, https://ebasis.eurofir.org/Default.asp, accessible on 6 June 2022). Currently, eBASIS contains 44,664 datapoints for bioactive compounds for 276 plant-based foods, distributed in main classes, e.g., 677 datapoints for phenols, 3945 datapoints for flavonols, 4581 datapoints for anthocyanins, 881 datapoints for carotenoids, 2695 datapoints for lignans, and 2654 datapoints for glucosinolates (https://ebasis.eurofir.org/Default.asp, accessed on 6 jube 2022).

Information included in eBASIS was described by Pilegaard et al. [70] and, in 2011, the utility of eBASIS tested in a phytosterols case study [71]. In 2017, a new interface linking the eBASIS bioactives database and the Creme Nutrition^®^ model was developed for the BACCHUS project (http://bacchus.cremeglobal.com/bacchus/, accessed on 26 October 2022) [72]. The eBASIS-Creme Global exposure tool enables users to assess compound intakes from various foods across populations to determine whether compounds required to obtain a claimed effect can be reasonably consumed within a balanced diet [72]. In 2018, an update on extractable and non-extractable antioxidants was completed [73] with the addition of 437 quality-evaluated datapoints. This update was the first example of building a resource dedicated to antioxidant properties within the existing resource. An updated eBASIS user guide was published at the same time, covering data concerning antioxidant properties and extractable and non-extractable compounds (https://ebasis.eurofir.org/files/basis_antiox.pdf, accessed on 6 June 2022).

The input form for data includes bibliographic references, food information (i.e., plant, part, subspecies/cultivar, maturity, season, growing conditions, etc.), processing (i.e., shape, state or form, heat treatment, cooking method, treatment applied, preservation method), sampling information (i.e., primary sample unit size, analytical sample size, sample plan, sample handling, etc.), compositional information (i.e., compound class, analytical method, concentration, extraction, and preparation, identification, etc.), and quality assessment. For each eBASIS section (plant/food description, processing defined, sampling plan, sample handling, compound identification, analytical method, analytical performance), transparent quality systems are included, ensuring eBASIS as a reliable resource for research with up-to-date information about plant food phytochemicals.

eBASIS was developed to present raw rather than aggregated data, reflecting variations in bioactive compositions related to cultivar, plant part, growing conditions, processing, and country of origin; there are multiple datapoints for each compound/food combination. To better meet requirements for aggregated bioactive composition data in dietary intake assessment, eBASIS data structures are being organized to link plant food data and bioactives with dietary intake assessment outputs and coding systems. At the same time, the architecture permits future inclusion of food data from animal origins and/or addition of new data on other plant foods/products or classes of compounds, emphasizing the need to envisage potential needs and gaps during development.

#### 2.2.3. ePlantLIBRA

In the area of dietary supplements (FDA definition)/food supplements (EFSA definition) [74], ePlantLIBRA [75,76] presents comprehensive and searchable data describing bioactive compounds specific to plant-based food supplements and botanicals, reporting health benefits, adverse effects, contaminants, and residues. ePlantLIBRA was developed by the PlantLIBRA project (PLANT food supplements: Levels of Intake, Benefit and Risk Assessment, Grant Agreement ID: ID: 245199) [77], which addressed development, validation, and dissemination of data and methodologies for risk and benefit assessment of plant food supplements and botanicals, and sustainable international cooperation in this domain [77].

ePlantLIBRA has the same structure as eBASIS; it is based on a user-friendly, efficient, and flexible interface for searching, extracting, and exporting data including links to the original references [76]. The architecture is based on eBASIS, MoniQA contaminant (FP6 Monitoring and Quality Assurance in the total food supply chain, Grant Agreement ID: 36337), and FERA’s HorizonScan databases (https://www.eurofir.org/our-tools/eplantlibra/, accessed on 26 October 2022). A webinar is available (https://www.eurofir.org/our-tools/eplantlibra/, accessed on 26 October 2022) with short videos covering the functionality of ePlantLIBRA (https://eplantlibra.eurofir.org/Default.asp, accessed on 6 June 2022).

Currently, 45,168 and 117 datapoints are available for composition and beneficial data, respectively, and 55 are specifically addressed to plant-based food supplements or botanicals, e.g., aloe vera extract, borage oil, pomegranate supplement, boswellia products, cinnamon products, dandelion products, and so on (https://eplantlibra.eurofir.org/Default.asp, accessed on 6 June 2022).

#### 2.2.4. FoodWasteExplorer

Advances in food research are increasingly directed towards sustainability of food chains, including exploitation of unconventional foods/waste for biologically active compounds, and reuse or recycling to achieve a circular economy. FoodWasteEXplorer [78] brings together the compositions of some of the most common products and their associated side streams and was developed within the EU-founded project REFRESH (REFRESH: Resource Efficient Food and dRink for the Entire Supply cHain, Grant Agreement ID: 641933, https://eu-refresh.org/, accessed on 6 June 2022). Currently, FoodWasteEXplorer contains 27,069 datapoints, including 587 nutrients, 698 bioactives, and 49 toxicants, gathered from peer-reviewed papers, grey literature (e.g., manufacturers’ data), and other sources (https://ws.eurofir.org/foodwasteexplorer/about, accessed on 6 June 2022). Food and side streams in FoodWasteEXplorer are searchable and grouped under areas of interest such as wine and beer, spirits, cider, cereals, chocolate, (fruit and vegetable) juices, cheese, animal products, sugar, vegetable oil, and coffee production. They are also grouped into food categories, e.g., cereals; milk and dairy; eggs; fats and oils, nuts and seeds; fish and seafood; fruits and vegetables; beverages; and other (i.e., algae, frog, snail, etc.). Finally, specific searchable functions—by foods, side streams, components—are available, e.g., by searching for foods, coffee, related side stream* information about the compositions of coffee grounds, coffee husks, coffee hulls, coffee leaves, coffee pulp (dried), coffee oil meal, malt coffee marc, instant coffee by product, and coffee parchment are described.

#### 2.2.5. Other Developing/Ongoing Resources: FoodCASE

FoodCASE was developed by Premotec GmbH (CH) in partnership with EuroFIR AISBL to manage food composition, food consumption, total diet study (TDS), laboratory food analysis, and branded food data, assembling food information in one system to promote re-use by linking food lists to other datasets and resources [79,80]. This data management system has wizards to support advanced data operations such as data import and export, recipe calculations, dataset linkage, nutrient estimation, data issue, and data quality analysis. It also supports different processes involved in the acquisition, management, and processing of data and uses European and international standards for the different datasets [80].

## 3. Ongoing Work and Future Directions

To ensure that EuroFIR AISBL resources remain valuable to user communities, it is important not only to update, expand, and enhance databases, but also to do these in standardized and harmonized ways among organizations and countries, considering existing and emerging food sources, and adding new descriptors and markers as necessary. To this end, engagement with networks and research infrastructures is a priority, creating synergies necessary to generate high-quality data and develop tools for the production, management, and exploitation of food data. In line with the European Strategy Forum on Research Infrastructures (ESFRI), the research infrastructure METROFOOD-RI and the European Open Science Cloud (EOSC), strategies leading to reliable and comparable analytical measurements in foods along food chains, from primary producers to consumers and beyond (food waste) and increasingly FAIR data [81] are valuable for researchers, food producers, and consumers. However, continued cooperation and sharing of data between compilers and users, within an integrated approach for agro-food, nutrition, and health, are key to success. Management of data at agro-food, nutrition, and health interfaces is a priority, but integrating FCDBs and infrastructures (interoperability) can only be achieved if approaches are applied based on metrological principles [81,82,83,84,85,86].

In this context, EuroFIR AISBL is involved in Member and Client activities and EU or otherwise-funded projects considering a range of relevant topics. The Food Nutrition Security Cloud (FNS-Cloud, Grant Agreement ID: 863059, www.fns-cloud.eu, accessed on 26 October 2022) aims to support integration of existing and emerging food research data and tools to address diet and health research questions across agro-food, nutrition and lifestyle, and non-communicable disease and healthy diet domains [87].

EuroFIR AISBL is also active in the proposed Food Nutrition Health Research Infrastructure (FNH-RI), which aims to link food production (agriculture and food technology) and food consumption (food determinants, intake, nutrition, and health) domains. To this end, a prototype *Determinants and Intake Platform*, harmonizing and linking consumer food behaviors, was formulated based on EuroDISH (Study on the need for food and health research infrastructures in Europe, Grant Agreement ID: 311788) and RICHFIELDS (Research infrastructure on consumer health and food intake using e-science with linked data sharing, Grant Agreement ID: 654280) outputs [88].

With the food environment undergoing vast changes, the need to study the nutritional variation in processed foods has driven an international move for branded food composition databases (BFCDBs). EuroFIR AISBL is working with its members to create a platform for collaboration and advocacy around BFCDBs, addressing user needs and gaps surveyed in 2020-2021. During the EuroFIR Food Forum 2021, a workshop was dedicated to BFCDBs, discussing advances at the European level and open access issues.

## Data Availability

Not applicable.
